# Experimental study on removing heavy metals from the municipal solid waste incineration fly ash with the modified electrokinetic remediation device

**DOI:** 10.1038/s41598-019-43844-w

**Published:** 2019-06-04

**Authors:** Hong Ji, Weiqiu Huang, Zhixiang Xing, Jiaqi Zuo, Zhuang Wang, Ke Yang

**Affiliations:** 1 School of Petroleum Engineering, Changzhou University, Changzhou, 213164 China; 2 School of Environmental and Safety Engineering, Changzhou University, Changzhou, 213164 China; 3 Jiangsu Key Laboratory of Oil-Gas Storage and Transportation Technology, Jiangsu, 213016 China

**Keywords:** Environmental sciences, Environmental sciences, Pollution remediation, Pollution remediation, Chemistry

## Abstract

The MSWI fly ash which contains a large number of heavy metal substances is a subsidiary product of waste incineration power generation technology. If the MSWI fly ash is disposed improperly, heavy metal pollutants will pose a great threat to environmental safety and human health. Based on the technology of electrokinetic remediation, the feasibility of removing heavy metal pollutants from the MSWI fly ash using a modified electrokinetic remediation device - cylinder device was evaluated in this study. Differing from the traditional cuboid device with the volume ratio of the cathode chamber to the anode chamber being 1:1, the volume ratio of the cathode chamber to the anode chamber of the cylinder device was 16:1. Changes in parameters, such as pH values and conductivity in the cathode and the anode chambers as well as current and voltage in the sample area were analysed under the voltage gradient of 2 V/cm. After the experiment, the average removal efficiencies for Zn, Pb, Cd and Cu in the sample area were 53.2%, 31.4%, 42.3% and 30.7%, respectively. It indicates that the cylinder device is effective in removing heavy metals from the MSWI fly ash. Adopting the cylinder device for the experimental study on the electrokinetic remediation technology could provide a better way of thinking for the future engineering practices and applications.

## Introduction

Along with global climate changes and economic development, most countries are suffering increasing pressure on resources, environment and ecology. Water and solid waste pollution problems are becoming more and more serious. Researchers have used different methods to handle water pollution problems, including electrochemical treatments, physicochemical processes, adsorption and other methods^1–14^. Meanwhile, properly disposing MSW has gradually become a hot issue of concern due to the large amount of municipal solid waste (MSW). As the best way to dispose MSW under the principle of “reduction, harmlessness and resource reuse”, the waste incineration power generation technology has drawn great attention from many countries^15–18^. The MSWI fly ash which contains a large number of heavy metal substances is a subsidiary product of waste incineration power generation technology. Failing to dispose the MSWI fly ash properly, secondary pollution will cause serious environment safety problems^19–24^.

Since the 1980s, researchers have used the electrokinetic remediation technology to remove pollutants from the soil. At present, the electrokinetic remediation technology has presented better prospects with respect to the environmental governance of heavy metal contaminated soil, industrial solid waste and MSWI fly ash^25–40^. Some studies suggested that the electrokinetic remediation technology could effectively remove heavy metal pollutants from the MSWI fly ash.

A cuboid remediation device is generally adopted in the traditional electrokinetic remediation technology, including an anode chamber, a sample area and a cathode chamber from left to right in order; of which, the volumes of the cathode chamber and the anode chamber were equal. In order to enhance the removal efficiency of the electrokinetic remediation technology, the traditional experimental device was modified and designed in a cylinder shape as a whole in this study; the anode chamber was located in the central ring area of the cylinder device; the cathode chamber was located in the outer ring area; and the sample area was located between the central ring and the outer ring. Compared with the traditional remediation device, the volume of the cathode chamber was expanded in order to facilitate the migration of heavy metals in the MSWI fly ash.

In this study, an electrokinetic remediation experiment was carried out with the application of a self-made cylinder device in order to obtain a high removal efficiency of heavy metals. During the experiment, changes in parameters regarding pH and conductivity of electrolyte solution in the anode chamber and the cathode chamber as well as current and voltage in the sample area were monitored in real time. The efficiency of the cylinder device for removing heavy metals from the MSWI fly ash was analysed after the experiment.

## Experimental Materials and Methods

### Materials

**Figure 1:**
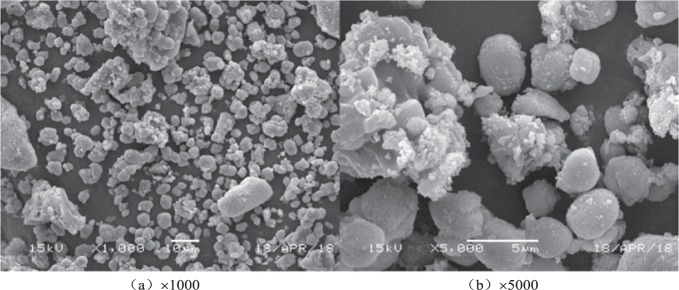
The MSWI fly ash in scanning electron microscope.

In this study, MSWI fly ash samples were collected from the ash discharge silo of the flue gas purification system in a waste incineration power plant in Yixing, Jiangsu province. MSWI fly ash samples were sieved through a sieve of 0.2 mm to remove large particles before being placed in a constant temperature heater to be dried at 110 °C. The specific element analysis in the MSWI fly ash samples was determined by XRF (XRF 1800CCDE), as shown in Table 1. The morphology of MSW fly ash was obtained by scanning electron microscope, as shown in Fig. 1. Water used in the experiment was distilled water.

**Table 1:** Elements in the MSWI fly ash (%).

Ca	O	Cl	Na
29.8876	25.7237	15.2524	6.2318
K	S	Mg	Fe
5.8660	3.5988	1.8704	2.1669
Zn	P	Ti	Pb
0.6402	0.8306	0.6209	0.2038
Cu	Br	Sr	Mn
0.0805	0.0744	0.0744	0.0053
Si	Al	Cd	
4.9959	1.6531	0.1183

According to the morphology analysis in Fig. 1, it shows that the fly ash is presented in an irregular flocculent distribution with loose structure and rough surface. Meanwhile, based on XRF monitoring results in Table 1, it’s obvious that the fly ash consists of O, Ca, Cl, Na, K, Si, S and other trace elements. Furthermore, heavy metal elements (such as Pb, Zn, Cd, Cr, Cu and Mn) exist in a form of trace elements; meanwhile, the content of Zn is higher than that of Pb, Cd and Cu.

### Experimental devices and experimental design

**Figure 2:**
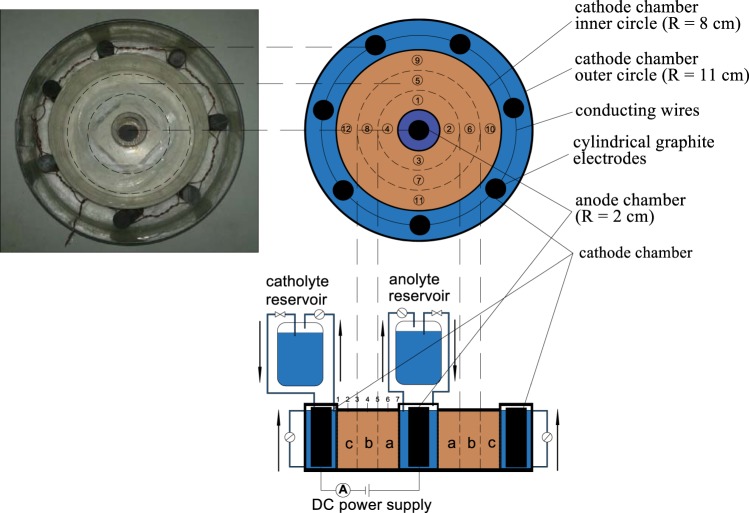
Electrokinetic remediation experimental set-up.

The experimental device was shown in Fig. 2. The modified electrokinetic remediation device is mainly composed of power supply, electrodes, electrode chambers (incl. the cathode chamber and the anode chamber) and a fly ash sample area. A DC power supply (NHWY60–250, Nover Power Co., Ltd. China) is adopted to provide constant voltage for the fly ash sample. The electrode chambers include the cathode chamber and the anode chamber with volumes of 19 ml and 312 ml, respectively; while the sample area with a volume of 377 mL is located between the cathode chamber and the anode chamber. The cylindrical graphite electrode with an underside radius of 5 mm and a height of 80 mm is adopted as the electrode in the electrode chambers. The cylindrical graphite electrode strips were placed in the anode chamber (n = 1) and the cathode chamber (n = 7), and the cylindrical graphite electrode strips were connected with the electrode chambers using silver conducting wires. The ring fence between the anode chamber and the sample area was wrapped with the quantitative filter paper, while the ring fence between the cathode chamber and the sample area was wrapped with the cation exchange membrane and the quantitative filter paper. The reason why the cation exchange membrane is wrapped is that the cation exchange membrane can prevent OH^−^ in the electrolyte of the cathode chamber from entering the sample area to the maximum extent. However, any cation can penetrate through the membrane. In this way, OH^−^ in the electrolyte of the cathode chamber can be prevented from the sample area. After that, 720 g prepared dried MSWI fly ash were put into the sample area of the experimental device and filled with 850 mL distilled water to 5 mm slightly higher above the fly ash surface. In order to measure the voltage, seven inert needle electrodes with the distance of 1 cm between each of them were inserted in the sample area. A four channel peristaltic pump (BT-M/4*YZIII, Baoding Zhunze Precision Pump Co., Ltd. China) was utilized to circulate electrolyte and control the liquid level in the device. The rotating speed of the peristaltic pump was 30 rpm and the flow rate of the liquid in the tube was 15 mL/min. After the fly ash was equilibrated for 24 hours, the pump was switched on immediately. Two channels of the pump and 2 valves started to work, adding electrolyte and controlling the liquid level of the cathode and anode chamber as well as the liquid level of the fly ash. The electrolyte in the cathode and the anode chamber was stirred in a circulatory manner with the other two channels of the peristaltic pump. The experiment was repeated three times to ensure the accuracy of the experimental data. Specific experimental parameters are shown in Table 2.

**Table 2:** Electrokinetic remediation experimental parameters.

Electrode Distance(cm)	Voltage(V)	Voltage gradient(v/cm)	Moisture Content(%)	Time(d)
6	12	2	100	15

During the experiment, the voltage of the sample area should be measured by divisions in the subareas of 2 cm (a-ring subarea) and 4 cm (b-ring subarea) and 6 cm (c-ring subarea) away from the outer circle of the anode chamber in the annulus sample area, respectively. Each circular ring was divided into four points for measuring (a ring is corresponding to measuring points 1, 2, 3 and 4; b ring is corresponding to measuring points 5, 6, 7 and 8; and c ring is corresponding to measuring points 9, 10, 11 and 12), respectively. At last, the average voltage was obtained from 4 measuring points in each ring.

After the experiment, the leaching toxicity of the fly ash samples was analysed. Each ring was divided equally into four points for sampling a ring is corresponding to sampling points 1, 2, 3 and 4; b ring is corresponding to sampling points 5, 6, 7 and 8; while c ring is corresponding to sampling points 9, 10, 11 and 12), respectively. Finally, the heavy metal contents of Pb, Zn, Cd and Cu were measured and the obtained values were averaged, respectively, as shown in Fig. 2.

### Leaching toxicity test and analysis method

In this study, leaching toxicity identification experiment of MSWI fly ash samples was carried out according to the “A leaching toxicity method-acetic acid buffered solution method” (HJ/T300-2007). Contents of Pb, Zn, Cd and Cu in the samples were determined by AAS (Analytik Jena Co., Ltd. Model novAA400). The detailed parameters are shown in Table 3.

**Table 3:** Experimental parameters of leaching toxicity identification.

Experiment method	Leaching agent	Liquid-solid ratio (L/Kg)	Rotational Speed (r/min)	Temperature (^0^C)	Oscillation time (h)
HJ/T300-2007	Acetic acid buffer solution	20:1	30 ± 2	23 ± 2	18 ± 2

The removal efficiency of heavy metal leaching toxicity was calculated as the ratio of the reduced part of heavy metal leaching toxicity value in the fly ash after electrokinetic remediation to the original heavy metal leaching toxicity value in the fly ash before electrokinetic remediation. Its mathematical expression can be presented as follows:

1 $$w=\frac{{C}_{0}-C}{{C}_{0}}\times 100 \% =(1-\frac{C}{{C}_{0}})\times 100 \% $$

Where, w is the efficiency of removing heavy metals; C_0_ is the original heavy metal leaching toxicity value in the fly ash; C is the heavy metal leaching toxicity value in the fly ash after the electrokinetic remediation; C/C_0_ is the ratio of leaching concentration, which could directly express the efficiency of removing heavy metals in different sections of the sample area before and after the electrokinetic remediation. All experiments were conducted in triplicate, and the average values were used in the data analysis.

## Results and Discussion

### Changes in pH values of electrolyte solution in the anode chamber and the cathode chamber

**Figure 3:**
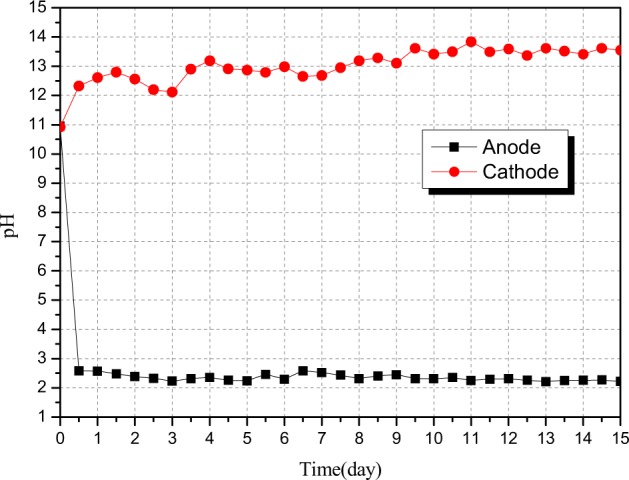
Changes in pH values of electrolyte solution in anode chamber and cathode chamber.

The curve graph of pH values of electrolyte solution in the anode chamber and the cathode chamber changing with time is presented. Changes in the initial pH values of the anode chamber and the cathode chamber were a sign of the cylinder electrokinetic remediation device entering the start-up stage. According to Fig. 3, it can be seen that the pH values of the electrolyte solution in the anode chamber and the cathode chamber at the initial time were 10.9, being alkaline. It was determined by the characteristics of the fly ash. In the experiment, it was found that the pH values of the anode chamber and the cathode chamber had changed significantly within 10 hours after the power was energized. Specifically, the pH value of the electrolyte solution in the anode chamber was dropped sharply to 2.58, while the pH value of the electrolyte solution in the cathode chamber was raised to 12.32, indicating that electrolytic reactions were conducted in the water of the anode chamber and the cathode chamber. That is, water molecules in the anode chamber were oxidized after the loss of electrons, forming oxygen and H^+^; while water molecules in the cathode chamber were reduced after the acquisition of electrons, forming hydrogen and OH^−^. The pH value of the electrolyte solution in the anode chamber was maintained steady after dropping to around 2.58 and fluctuated slightly around 2.58. The pH value of the electrolyte solution in the cathode chamber was maintained steady after rising to 12.32 within the first 10 hours of the experiment with a fluctuation between 12.3 and 13.5. The pH change of electrolyte solution in cathode chamber was mainly affected by heavy metal cations leached in the sample area. The heavy metal cations would form hydroxide precipitation with OH^−^ when it’s penetrated through the cation exchange membrane to the cathode chamber^26^. Thus, parts of the OH^−^ were consumed. When the forming rate of OH^−^ was higher than the consuming rate of heavy metal ions on the OH^−^, the pH value of electrolyte solution in the cathode chamber may be increased slightly.

### Changes in conductivities of the electrolyte solution in the anode chamber and the cathode chamber

**Figure 4:**
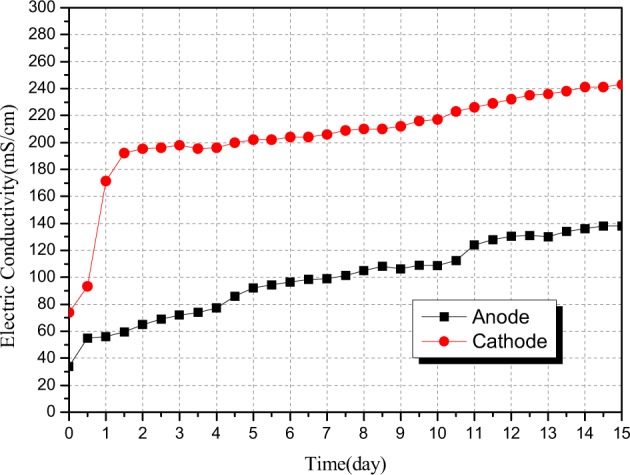
Changes in conductivity of electrolyte solution in the anode chamber and the cathode chamber with time.

As shown in Fig. 4, the overall conductivity of the electrolyte solution in the anode chamber and the cathode chamber is presented in an increasing trend. There are two contributors to the slow rise of the conductivity of the electrolyte solution in the anode chamber; on the one hand, a large amount of H^+^ ions were produced by water electrolysis in the anode chamber; on the other hand, the dissociation of soluble compounds in the sample area produced a large number of anions (such as PO_4_^3−^, H_2_PO_4_^−^, HPO_4_^2−^, Cl^−^, CO_3_^2−^, SiO_3_^2−^, etc.) along with the electrokinetic remediation experiment, and the anions migrated towards the anode chamber with different electrical properties under the action of electric field, and finally entered the anode chamber. The experimental results indicated that the H^+^ ions produced by water electrolysis in the anode chamber were released under the action of electric field to carry out relocation diffusion towards the sample area and the cathode chamber. In principle, the concentration of H^+^ in the anode chamber will be decreased, thus decreasing the conductivity of electrolyte solution in the anode chamber. In fact, the conductivity was increased constantly, since a large number of anions have been migrated to the anode chamber constantly. In the process of electrokinetic remediation experiment, heavy metal elements in the sample area were released in the form of cations before the continuous spread towards the cathode chamber. Adding a cation exchange membrane between the sample area and the cathode chamber can effectively prevent heavy metal cations from combining with OH^−^ ions in the sample area to form precipitation, so that the heavy metal ions could be migrated to the cathode chamber under the action of electric field to be precipitated in the cathode chamber and be adsorbed on the graphite electrode. Thus, the heavy metal elements in the fly ash samples could be removed. Water electrolysis in the cathode chamber produced a large number of OH^−^ ions which would easily produce hydroxide precipitations with the heavy metal cations migrated from the sample area, so that the produced OH^−^ ions would be consumed due to the reaction with the heavy metal cations. Theoretically, the conductivity of the cathode chamber should be reduced; but the observed experimental data suggested that the conductivity of the electrolyte solution in the cathode chamber is steady with a small increasing trend in the middle and the late stages of the experiment. Possible explanations for these results may be that: on the one hand, the rate of electrolysis producing OH^−^ ions was higher than the rate of heavy metal cations consuming OH^−^ ions; on the other hand, heavy metal elements such as Pb and Zn, presenting in the states of [Pb(OH)_3_]^−^, [Pb(OH)_4_]^2−^, [Zn(OH)_3_]^−^ and [Zn(OH)_4_]^2−^ would may also lead to the increase of conductivity.

### Changes in current in the sample area

**Figure 5:**
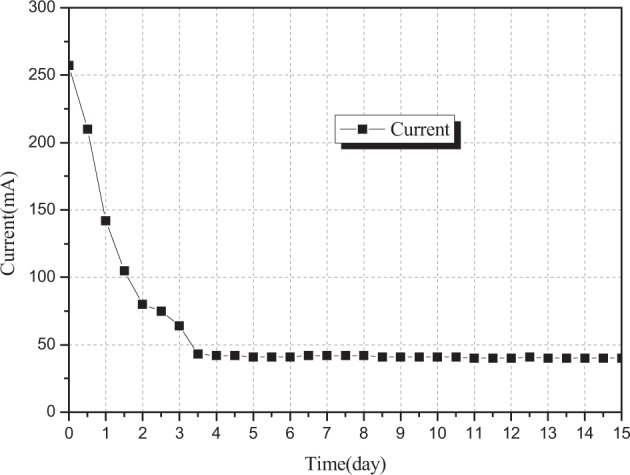
Changes in current in the sample area over time.

Figure 5 shows the changes in current in the sample area over time during the experiment on the cylinder device. The magnitude of the current in the sample area was positively correlated with the amount of electric charge passing through the fixed interface area (in the pore solution of fly ash) within unit time, with the opposite direction of the motion of the electrons. At the same time, the current was also an important factor affecting the removal efficiency for heavy metals^28^.

Under the voltage gradient of 2 V/cm, the current reached the peak value of 257.2 mA within 2 hours; and 3 days later, the current was stable and maintained about 40 mA, as shown in Fig. 4. The same change rule was also reported in the electrokinetic remediation experiment of soil pollutants^34,41^. In the process of electrokinetic remediation for removing heavy metals from the fly ash, the current was closely related to the conductivity which was related to the concentration of ions in the pore solution. The larger the ion concentration, the greater the current will be in the sample area. The main types of ions capable of moving freely in this system included: (1) H^+^ ions produced in the anode chamber and OH^−^ ions produced in the cathode chamber after water electrolysis; (2) ions produced by complete ionization of electrolyte solution added in the anode chamber; and (3) cations and anions produced by complete ionization of soluble salts without containing heavy metals (calcium chloride, potassium chloride, etc.), cations and anions produced by complete ionization of soluble salts containing heavy metals (including cadmium chloride, zinc chloride, copper chloride, etc.), and cations and anions produced by dissociation of insoluble or indissoluble salts or other complex compounds containing heavy metals (basic zinc chloride, basic lead carbonate, basic copper carbonate, zinc carbonate, zinc oxide, zinc silicate, etc.) under acidic conditions. At the beginning of the experiment, various cations and anions moved towards the anode chamber and the cathode chamber. Gradually, different kinds of ions were leached from the pore solution of fly ash over time. Under the action of electromigration and electroosmotic flow, the leached cations and anions were migrated and enriched in the anode chamber and the cathode chamber, respectively^42–45^. Moreover, insoluble or non-electric compounds were produced by cations in the cathode chamber together with OH^−^ ions, which were eventually attached to the surface of the cathode graphite electrode to form protective oxidation film, passivation film or other insoluble corrosion materials, resulting in the increased resistance and decreased current in the sample area^31,46^.

According to the Ohm law, higher applied voltage would be associated with larger current in the conducting medium if medium of ions during migration had the same resistivity during the migration^32^. However, due to the effectiveness of the ions in the experiment, the pH values and chemical compositions of the pore fluid varied in time and space with the electrokinetic remediation experiment. As the resistance of the fly ash samples was constantly changing, the current in the sample area was stable and maintained about 40 mA instead of obeying the Ohm law in most of the experimental time range.

### Changes in voltage distribution in each section of the sample area

**Figure 6:**
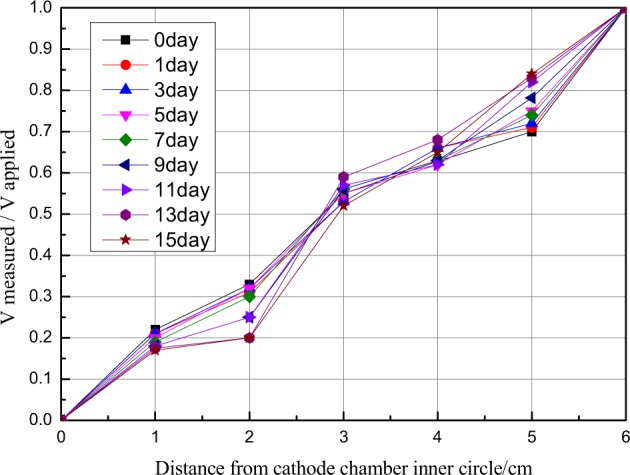
Changes of voltage in each section of the sample area over time.

Changes in voltage distribution in the fly ash sample area during the 15 days of electrokinetic remediation at 2.0 V/cm are displayed in Fig. 6. Because the applied potential difference (12 V) between the cathode graphite electrode and the anode graphite electrode was constant during the experiment and the fly ash sample area was actually non-conductive, voltage of the fly ash sample area was inversely proportional to the relative conductivity of the pore solution^47^. The ratio of test potential to applied potential and the distance from the cathodes in the fly ash sample area was almost in linear relation, indicating that the ion composition of the fly ash pore solution in the sample area was relatively stable. The higher potential gradient in the fly ash area near the two electrodes may be related to the high resistance of the solid-liquid interface of the electrodes^48^. Ion migration in the pore solution became an important factor determining the voltage distribution in a long period of electrokinetic treatment. The voltage gradient caused not only the migration of ions to electrodes with opposite charges, but also lead to the flow of the pore solution. After the experiment, under the combined action of electromigration and electroosmotic flow, the number of ions in the pore solution near the cathode area exceeded the number of ions in the pore solution near the anode area, that is, the conductivity of fly ash near the cathode area was higher than that in the anode area^43–45^. As shown in Fig. 6, voltage near the cathode area decreased slowly with the increase of the remediation time, while voltage near the anode area increased slowly with the increase of the remediation time.

### Removal efficiency for heavy metals after electrokinetic remediation

**Figure 7:**
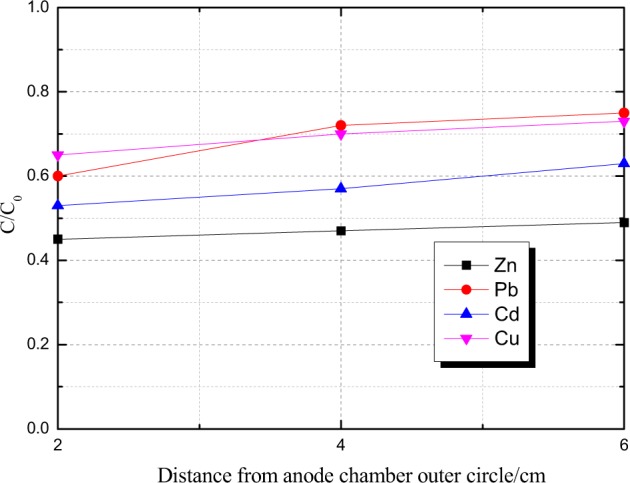
The ratio of the leaching concentration of heavy metals after electrokinetic remediation experiment to the leaching concentration of heavy metals in the original fly ash.

Distribution of the ratio of the leaching concentration of heavy metals in each section of the sample area to the concentration of heavy metals in the original fly ash after the experiment is described in Fig. 7. C/C_0_ values of each sampling section showed a small fluctuation as a whole. Furthermore, the C/C_0_ value in the Section a near the anode area was smaller than that in the Section c near the cathode area. In addition, the efficiency of removing Zn, Pb, Cd and Cu in the Section a near the anode chamber was obviously better, which was similar to the traditional cuboid device. This may be caused by the situation that the Section a near the anode chamber was directly influenced by the H^+^ migrating from the anode chamber, while the leaching rate of heavy metals in acidic conditions was slightly higher than that in the other sections. Overall, the C/C_0_ values of various heavy metals ranged from 0.4 to 0.8, directly reflected the efficiency of removing heavy metals after the electrokinetic remediation experiment. That is, the higher the C/C_0_ value, the lower the removing efficiency will be, and vice versa. After the experiment, the average removing efficiencies of Zn, Pb, Cd and Cu were 53.2%, 31.4%, 42.3% and 30.7%, respectively. The pH value changed smoothly with small amplitude of oscillation in general in cathode and anode compartments except the initial break. The electrical current rapidly increased on the first day of the experiment and steadily declined after that and the electrical conductivity presented a clear rising trend. The residual partition of detoxified samples were obviously lifted to which was much higher than the analysis data of the raw materials. The pH and the electrical conductivity in sample region were distributed more uniformly and the blind area was effectively eliminated in the electrolytic cells, which were indirectly validated by the contrastive detoxification result of the spiked HMs between the rectangular and cylindrical devices. Compared with the traditional cuboid device, a large number of white flocculent sediments were not observed in the Section C near the cathode groove, which indicates that the design of the cylindrical device can effectively prevent the OH^−^ ions produced by the cathode groove from escaping to the sample area, thus avoiding the formation of flocculent precipitation by heavy metal cations reacting with OH^−^ ions.

## Conclusions

In this thesis, an experimental study on the electrokinetic remediation of MSWI fly ash was carried out with the use of the cylinder structure. Changes in initial pH values of the two electrode chambers were considered as signs for determining whether the cylinder device enters the start-up state. The magnitude of the solution conductivity in the electrode chambers could reflect the capacity of the electrolyte solution for electric conduction. In view of the whole process of electrokinetic remediation, the conductivity of the two electrode chambers showed a slow upward trend during the experiment. The magnitude of the current in the sample area was positively related to the ion concentration in the pore solution of fly ash, namely, the larger the ion concentration, the greater the current would be in the sample area. Ions migrated to electrodes with opposite charges, causing the flow of pore solution due to the voltage gradient. Under the combined action of electromigration and electroosmotic flow, the ion concentration in the pore solution near the cathode area was higher than that in the pore solution near the anode area. Meanwhile, the conductivity of fly ash in the cathode area was higher than that in the anode area.

After the experiment, the average efficiency of removing heavy metals from fly ash for the cylinder device was analysed. The average removing efficiencies for Zn, Pb, Cd and Cu were 53.2%, 31.4%, 42.3% and 30.7%, respectively. In sum, test results suggested that the cylinder experimental device has a certain superiority. The experimental study on electrokinetic remediation technology carried out with the use of cylinder experimental device will provide a better way of thinking for the future engineering practices and applications.
